# Antibacterial activity of plant-extract mediated silver nanoparticles against *Klebsiella* spp. in Africa: a systematic review

**DOI:** 10.3389/fmicb.2025.1673235

**Published:** 2026-01-12

**Authors:** Marcus Barlay Dunah, Mai Ali Abdalla, Ibrahim G. Wawata, Tijani Naheem Adekilekun

**Affiliations:** 1 Department of Microbiology and Immunology, Kampala International University, Ishaka-Bushenyi, Uganda; 2 Department of Pharmacy, Kampala International University, Ishaka-Bushenyi, Uganda

**Keywords:** antibacterial activity, green synthesis, silver nanoparticles (AgNPs), geographical distribution, nanobiotechnology, plant extracts, systematic review, phytochemical reduction

## Abstract

**Introduction:**

Antimicrobial resistance (AMR) among *Klebsiella* spp. is an escalating global health concern, particularly in resource-limited settings. The rapid spread of multidrug-resistant strains has rendered conventional antibiotics increasingly ineffective. In Africa, the green synthesis of silver nanoparticles (AgNPs) using medicinal plant extracts offers a sustainable and cost-effective approach for developing novel antibacterial agents.

**Methods:**

This systematic review followed PRISMA 2020 guidelines. A comprehensive search was conducted across PubMed, Scopus, Web of Science, Google Scholar, and African Journals Online (AJOL) for studies published between January 2015 and May 2025. Eligible studies included *in vitro* experiments evaluating the antibacterial activity of plant extract–mediated AgNPs against *Klebsiella* spp. Two reviewers independently screened articles, extracted data, and assessed quality using the Joanna Briggs Institute (JBI) Critical Appraisal Checklist for *in vitro* studies.

**Results:**

Out of 323 identified records, 14 studies met the inclusion criteria. The studies originated from Egypt (*n* = 4), Nigeria (*n* = 4), Kenya (*n* = 3), South Africa (*n* = 2), Tunisia (*n* = 1), and Ghana (*n* = 1). Frequently used plant species included *Azadirachta indica, Moringa oleifera, Vernonia amygdalina, Allium sativum, Carica papaya,* and *Ocimum gratissimum*. Silver nanoparticles synthesized from these extracts were typically spherical and measured 20–100 nm in diameter. All studies reported significant *in vitro* antibacterial activity, with zones of inhibition ranging from 10 to 24 mm and minimum inhibitory concentrations (MICs) ranging from 6.25 to 50 μg/mL.

**Conclusion:**

Plant-mediated silver nanoparticles exhibit strong *in vitro* efficacy against *Klebsiella* spp., supporting their potential role as alternative or adjunct antimicrobial agents in managing AMR. However, further *in vivo* studies, toxicological assessments, and standardization of nanoparticle synthesis and testing protocols are essential for their translation into clinical application.

## Introduction

Antimicrobial resistance (AMR) constitutes a significant and growing threat to global health, affecting both low- and high-income countries alike (World Health Organization (WHO), 2017; Zhou et al., 2023). Among the most problematic pathogens are members of the genus *Klebsiella*, particularly *Klebsiella pneumoniae*, which frequently cause hospital-acquired infections, pneumonia, septicemia, and UTIs, and exhibit resistance to multiple antibiotic classes (Rahman et al., 2024; Navon et al., 2021). This resistance is primarily driven by the extensive use of antibiotics and the horizontal transfer of resistance genes, fueling the alarming proliferation of multidrug-resistant (MDR) strains (Marambio-Jones and Hoek, 2010).

In Africa, the problem of antimicrobial resistance is exacerbated by limited healthcare resources, poor antibiotic stewardship, inadequate surveillance, and delayed diagnostics (World Health Organization (WHO), 2017; Founou et al., 2021a,b). Therefore, there is an urgent need for alternative and sustainable strategies to combat these resilient pathogens (Durán et al., 2016). Traditional medicine, which utilizes plant-derived compounds, offers a rich reservoir of bioactive materials with potent antimicrobial properties.

Green synthesis of silver nanoparticles (AgNPs) using plant extracts has recently emerged as a promising approach for developing new antimicrobial agents (Negri et al., 2021; Otun et al., 2021). This method utilizes phytochemicals as reducing, stabilizing, and capping agents, thereby avoiding toxic chemicals and reducing production costs (Negri et al., 2021; Otun et al., 2021). Metallic nanoparticles have fascinated scientists for over a century and are now heavily utilized in biomedical sciences and engineering (Morante et al., 2024). They are a focus of interest because of their huge potential in nanotechnology. Today, these materials can be synthesized and modified with various chemical functional groups, which allow them to be conjugated with antibodies, ligands, and drugs of interest, thus opening a wide range of potential applications in biotechnology, magnetic separation, and preconcentration of target analytes, targeted drug delivery, and vehicles for gene and drug delivery and, more importantly, diagnostic imaging (Marambio-Jones and Hoek, 2010).

Evidence from numerous *in vitro* studies suggests that AgNPs can effectively inhibit the growth of both gram-positive and gram-negative pathogens, including *Klebsiella* spp. (Negri et al., 2021; Otun et al., 2021). The mechanisms by which AgNPs undermine microbial viability are multifaceted, involving disruption of microbial membranes, generation of reactive oxygen species, interference with metabolic pathways, and eventual cell death (Franci et al., 2015). Importantly, green synthesis may aid in reducing the potential for resistance development while preserving biocompatibility (Negri et al., 2021).

In recent years, green synthesis of silver nanoparticles using plant extracts has gained considerable attention as a promising strategy to combat antimicrobial resistance (AMR), particularly in resource-limited settings. In Africa, this approach has been increasingly explored due to its alignment with the continent’s rich ethnobotanical heritage and the urgent need for affordable, scalable alternatives to conventional antibiotics. Several studies conducted across countries such as Egypt, Nigeria, South Africa, and Kenya have demonstrated that phytosynthesized AgNPs exhibit significant *in vitro* antibacterial activity against multidrug-resistant pathogens, including *Klebsiella pneumoniae* (Otun et al., 2021; Ahmed et al., 2020; Adebayo et al., 2023; Njoroge et al., 2024). These successes reflect not only the antimicrobial efficacy of such nanoparticles but also the growing scientific capacity within African institutions to synthesize, characterize, and evaluate nanomaterials using indigenous plant species (Kibet et al., 2019). Although most applications remain at the experimental level, the accumulated data underscore the potential of green nanotechnology to serve as a regionally adaptable solution for AMR, with future success hinging on *in vivo* validation, standardization, and regulatory support.

*Warburgia ugandensis* (East African greenheart) remains one of the most widely used medicinal plants in East and Central Africa. Its bark, leaves, roots, and seeds are used traditionally to treat malaria, coughs, gastrointestinal disorders, toothache, fever, and skin diseases. Recent studies confirm its extracts show antibacterial, antifungal, antiplasmodial, antiviral, and anticancer properties (Musyoka et al., 2020; Omwenga et al., 2021; Adongo et al., 2023).

The bioactive drimane-type sesquiterpenes warburganal, polygodial, muzigadial, and drimenol have been identified as the main compounds responsible for antimicrobial and antiparasitic effects (Okello et al., 2020; Njogu et al., 2022). For instance, *warburganal* has shown strong antifungal effects against *Candida albicans* and Cryptococcus spp. (Omwenga et al., 2021).

Phytochemical analyses between 2020 and 2025 confirm the presence of more than 60 compounds, including sesquiterpenes, flavonoids, lignans, and fatty acids, many of which demonstrate cytotoxic, anti-inflammatory, and antioxidant properties (Musyoka et al., 2020; Njogu et al., 2022; Adongo et al., 2023). These compounds make the plant a strong candidate for drug discovery and novel therapeutic agents.

The species continues to play an important role in traditional healing practices across East Africa, where it is associated with protection and purification rituals (Omwenga et al., 2021). Economically, it supports the local herbal medicine trade and provides durable wood for construction and furniture. Its leaves are sometimes used as a food spice due to their peppery taste (Okello et al., 2020).

As an indigenous species, *W. ugandensis* contributes to agroforestry systems, providing shade, protecting against soil erosion, and supporting biodiversity. Its role in mixed-farming systems highlights its ecological sustainability value (Njogu et al., 2022).

Despite its medicinal and economic value, *W. ugandensis* faces severe threats from overharvesting, especially unsustainable bark stripping. It is currently listed as Vulnerable on the IUCN Red List. Conservation strategies emphasize domestication, propagation through cuttings and tissue culture, and community awareness for sustainable harvesting (Musyoka et al., 2020; Adongo et al., 2023).

This systematic review aims to comprehensively evaluate the antibacterial activity of plant extract-mediated silver nanoparticles against *Klebsiella* spp. in Africa. Specifically, we consolidate and analyze the available *in vitro* data to determine their efficacy, minimum inhibitory concentrations, and zones of inhibition, to guide future applications and further translational research in antimicrobial therapy.

### General objective

- To evaluate the antibacterial efficacy of Plant-mediated silver nanoparticles against *Klebsiella* spp. In Africa, to identify whether *Warburgia ugandensis* has been previously utilized in this context.

### Specific objective

1. To systematically identify African studies from 2015 to 2025 that assessed the antibacterial activity of plant-extract mediated AgNPs against *Klebsiella* spp.
2. To analyze the synthesis methods, plant species used, and antibacterial efficacy (zones of inhibition, MIC/MBC values) reported in those studies.
3. To determine whether *Warburgia ugandensis* has been utilized in the biosynthesis of AgNPs for antimicrobial testing against *Klebsiella* spp.
4. To highlight promising plant species and nanoparticle characteristics that show strong anti-*Klebsiella* activity.
5. To identify limitations and areas for further research, including potential for underutilized medicinal plants such as *Warburgia ugandensis.*

### Research question

1. Has *Warburgia ugandensis* been used in the green synthesis of silver nanoparticles for antibacterial testing against *Klebsiella* spp. in Africa?
2. Which medicinal plants have been most frequently used in the biosynthesis of silver nanoparticles effective against *Klebsiella* spp., and how consistent are their reported antibacterial outcomes in Africa?
3. What are the predominant synthesis and characterization methods used for plant-extract-mediated silver nanoparticles in the included African studies, and how do this influence antibacterial efficacy?
4. What is the range and distribution of MIC, MBC, and zone of inhibition values reported for phytosynthesized AgNPs against *Klebsiella* spp. across different African regions?
5. What are the major limitations identified in current *in vitro* studies on plant-mediated AgNPs against *Klebsiella* spp., and how can future research improve standardization, safety assessment, and clinical relevance?

## Methods

### Study design

This systematic review was conducted in accordance with the Preferred Reporting Items for Systematic Reviews and Meta-Analyses (PRISMA 2020) guidelines (Page et al., 2021). The objective was to systematically identify, critically evaluate, and descriptively synthesize in vitro evidence on the antibacterial activity of plant extract–mediated silver nanoparticles (AgNPs) against *Klebsiella* spp. across African countries.

### Search strategy

A comprehensive literature search was performed using five electronic databases: PubMed, Scopus, Web of Science, Google Scholar, and African Journals Online (AJOL). The search covered studies published from January 2015 to May 2025 and was limited to articles written in English.

The search strategy employed a combination of free-text keywords and Medical Subject Headings (MeSH) using Boolean operators (AND/OR). Keywords included: “antibacterial activity,” “green synthesis,” “silver nanoparticles,” “plant extract,” “*Klebsiella*,” and “Africa.” Google Scholar was used to retrieve additional regionally published and grey literature not indexed in traditional repositories. The first 200 results sorted by relevance were manually screened.

Additionally, the reference lists of all eligible full-text studies were reviewed to capture any additional articles not identified through database searches.

### Eligibility criteria

Studies were included if they met the following criteria: 1. Investigated the antibacterial activity of plant extract–mediated silver nanoparticles against *Klebsiella* spp.; 2. Employed *in vitro* antimicrobial assays (disk diffusion, minimum inhibitory concentration [MIC], and/or minimum bactericidal concentration [MBC]); 3. They were conducted in at least one African country, 4. Were published in a peer-reviewed journal or regionally indexed repository; 5. Were published between 2015 and 2025; 6. They were written in English. Studies were excluded if they were reviews, editorials, case reports, conference abstracts, or lacked essential methodological details (e.g., nanoparticle synthesis protocol, bacterial strain tested, or assay used).

### Study selection

**Figure 1:**
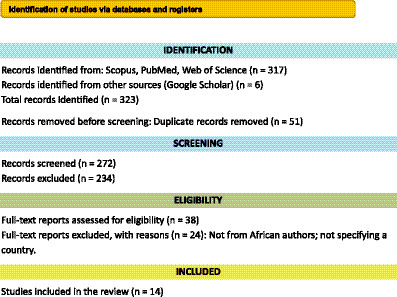
PRISMA flow diagram 2020.

Titles and abstracts retrieved from database searches were screened independently by two reviewers (MD and MA). Full-text articles of potentially eligible studies were then reviewed for inclusion against the eligibility criteria. Disagreements were resolved through discussion or, when needed, consultation with a third reviewer (IW). A total of 14 studies met all inclusion criteria and were included in the final synthesis, of which 3 were sourced exclusively from Google Scholar. The full selection process is illustrated in the PRISMA 2020 flow diagram (Figure 1).

### Data extraction

Data were extracted independently by two reviewers using a pre-defined and pilot-tested data extraction form. The following information was collected: Author(s), year of publication, country of origin, Plant species used for AgNP synthesis, Nanoparticle synthesis and characterization methods (e.g., UV–Vis, FTIR, SEM, TEM, XRD), *Klebsiella* strain tested (e.g., *K. pneumoniae*) Type of antimicrobial assay (disk diffusion, MIC, MBC), Quantitative outcomes (inhibition zone in mm, MIC/MBC in μg/mL), Summary of key findings and conclusions, Data discrepancies were resolved through joint discussion, Where information was unclear or missing, attempts were made to contact the study authors. However, the review did not focus on native medicinal plants in Africa, but rather on plants that had been used for the synthesis of silver nanoparticles in Africa.

### Quality assessment

Methodological quality and risk of bias were evaluated using the Joanna Briggs Institute (JBI) Critical Appraisal Checklist for Experimental (*in Vitro*) Studies (Mcarthur et al., 2020). This tool assesses eight key criteria: 1. Clarity of study objectives, 2. Appropriateness of study design, 3. Adequacy of nanoparticle synthesis and plant extract reporting, 4. Completeness of nanoparticle characterization, 5. Use of appropriate control groups, 6. Standardization of antimicrobial testing methods, 7. Reproducibility and clarity of results, and 8. Sufficient methodological detail to allow replication. Each study was assigned a risk rating of low, moderate, or high. Ratings were independently assigned by two reviewers and reconciled by consensus. The full scoring matrix is provided in Supplementary Tables.

### Data synthesis and descriptive statistics

Due to significant heterogeneity in plant species used, nanoparticle synthesis methods, antimicrobial assays, and reporting formats, meta-analysis was not feasible. Instead, a narrative and descriptive statistical synthesis was performed. Summary data were compiled into structured tables. Descriptive statistics were calculated for key parameters across the 14 included studies:

- Mean inhibition zone: 18.2 mm (range: 10–26 mm)
- Mean MIC value: 18.6 μg/mL (range: 3.1–50 μg/mL)
- Most common plant species: *Azadirachta indica, Vernonia amygdalina,* and *Moringa oleifera*
- Targeted strain: *Klebsiella pneumonia.*

Outcomes were stratified by geographic region, plant species, nanoparticle size, and type of antimicrobial assay. Patterns in efficacy were identified and discussed about methodological quality and synthesis parameters.

## Results

### Study selection

The systematic search yielded 323 records from five databases: PubMed, Scopus, Web of Science, Google Scholar, and AJOL. After removing 51 duplicates, 272 records were screened by title and abstract. Of these, 234 were excluded for irrelevance or methodological insufficiency.

A total of 38 full-text articles were assessed, and 14 studies met all eligibility criteria and were included in the final synthesis. These comprised 11 studies from indexed databases and 3 from Google Scholar. The selection process is outlined in the PRISMA 2020 flow diagram (Figure 1).

### General characteristics of included studies

The systematic search identified 323 records; after screening, 14 studies (2015–2025) met the inclusion criteria. These studies were conducted across six African countries: Egypt (*n* = 2), Nigeria (*n* = 5), Kenya (*n* = 3), South Africa (*n* = 2), Tunisia (*n* = 1), and Ghana (*n* = 1). All investigations employed plant extract–mediated green synthesis of silver nanoparticles (AgNPs) for antibacterial testing against *Klebsiella* spp.

### Plant species utilized in AgNP synthesis

A total of 12 plant species were reported across the included studies (Table 1). The most frequently investigated plants were:

- *Azadirachta indica* (*n* = 3)
- *Vernonia amygdalina* (*n* = 3)
- *Moringa oleifera* (*n* = 2)
- *Carica paaya* (*n* = 2)
- Other species used once included *Allium sativum*, *Ocimum gratissimum*, *Senna alata*, *Euphorbia hirta*, *Albizia coriaria*, *Mondia whitei*, *Tithonia diversifolia*, and *Harpagophytum procumbens*.

**Table 1:** Summary of plant species used for AgNP synthesis against *Klebsiella* spp. in Africa (2015–2025).

Plant species (Family)	No. of studies (n)	Countries	Nanoparticle size range (nm)	Typical antibacterial outcomes (Zone of inhibition/MIC/MBC)
*Azadirachta indica* (Meliaceae)	3	Egypt, Kenya	20–50	18–21 mm; MIC 6.25–12.5 μg/mL; MBC 12.5 μg/mL
*Vernonia amygdalina* (Asteraceae)	3	Nigeria, South Africa, Kenya	30–60	14–20 mm; MIC 12.5 μg/mL; MBC 25 μg/mL
*Moringa oleifera* (Moringaceae)	2	Egypt, South Africa	30–50	15–19 mm; MIC 6.25–12.5 μg/mL; MBC 12.5–25 μg/mL
*Carica papaya* (Caricaceae)	2	Egypt, Nigeria	40–100	10–22 mm; MIC 6.25–50 μg/mL; MBC 12.5–100 μg/mL
*Allium sativum* (Amaryllidaceae)	2	Tunisia, Kenya	20–100	10–20 mm; MIC 12.5–25 μg/mL; MBC 25–50 μg/mL
*Ocimum gratissimum* (Lamiaceae)	1	Ghana	30–50	22 mm; MIC 18.75 μg/mL; MBC not reported
*Senna alata* (Fabaceae)	1	Kenya	25–55	19 mm; MIC 6.25 μg/mL; MBC 12.5 μg/mL
*Euphorbia hirta* (Euphorbiaceae)	1	Nigeria	30–60	16 mm; MIC 12.5 μg/mL; MBC not reported
*Albizia coriaria* (Fabaceae)	1	Egypt	NR	20 mm; MIC 25 μg/mL; MBC not reported
*Mondia whitei* (Apocynaceae)	1	Kenya	NR	18 mm; MIC 10 μg/mL; MBC not reported
*Tithonia diversifolia* (Asteraceae)	1	Nigeria	NR	15 mm; MIC 25 μg/mL; MBC 50 μg/mL
*Harpagophytum procumbens* (Pedaliaceae)	1	Nigeria	NR	14 mm; MIC 22 μg/mL; MBC not reported

MIC, Minimum Inhibitory Concentration; MBC, Minimum Bactericidal Concentration; NR, Not reported.

Phytochemicals such as flavonoids, terpenes, alkaloids, and glycosides acted as reducing and stabilizing agents in nanoparticle formation. Plants rich in phenolics (e.g., *Azadirachta indica*, *Carica papaya*) consistently produced smaller nanoparticles and more potent antibacterial activity.y.

### Regional distribution of studies

The studies reflected regional biodiversity and research priorities (Figure 1). North Africa (Egypt, Tunisia, *n* = 5): Primarily used *Azadirachta indica*, *Carica papaya*, and *Allium sativum*, with well-structured characterization methods. West Africa (Nigeria, Ghana, *n* = 5): Dominated by *Vernonia amygdalina*, *Ocimum gratissimum*, and *Euphorbia hirta*. Most studies demonstrated high antibacterial efficacy, but with methodological variability. East Africa (Kenya, Uganda, *n* = 3): Used *Senna alata*, *Mondia whitei*, and *Aloe vera*. Ugandan data were limited but promising. Southern Africa (South Africa, *n* = 2): Applied *Moringa oleifera* and *Vernonia amygdalina*, with advanced nanoparticle characterization (UV–Vis, SEM, TEM, XRD).

### Nanoparticle synthesis and characterization

All studies employed aqueous plant extracts for green synthesis of AgNPs. Common characterization techniques included:

- UV–Vis spectroscopy (all 14 studies)
- FTIR analysis (10 studies)
- SEM/TEM imaging (9 studies)
- XRD (6 studies)

Particle sizes ranged from 20 to 100 nm, with most being spherical. Smaller particles (<50 nm) were associated with stronger antibacterial activity, particularly MIC ≤12.5 μg/mL.

### Antibacterial activity against *Klebsiella* spp.

All included studies demonstrated measurable *in vitro* antibacterial activity.

- Inhibition zones: 10–24 mm (mean: 18.2 mm).
- MIC values: 6.25–50 μg/mL (57% of studies reported MIC ≤12.5 μg/mL).
- MBC values: 12.5–100 μg/mL (reported in 5 studies).

Potent outcomes were consistently reported from AgNPs synthesized using *Azadirachta indica*, *Carica papaya*, and *Ocimum gratissimum*. Larger MIC values (>25 μg/mL) were observed in studies using *Tithonia diversifolia* and *Harpagophytum procumbens*.

### Target strains

Most studies (*n* = 11) specifically tested *K. pneumoniae*, while 3 reported only “*Klebsiella* spp.” without further specification. No study investigated resistant genotypes (e.g., ESBL- or carbapenemase-producing strains), highlighting a key research gap.

### Antibacterial mechanism of action

Evidence from the included studies indicated that green-synthesized AgNPs exert antibacterial effects through multiple synergistic mechanisms, including:

- Disruption of bacterial membrane integrity, leading to increased permeability and cell lysis
- Generation of reactive oxygen species (ROS), causing oxidative stress and damage to intracellular components
- Binding to proteins and DNA, interfering with replication, enzyme activity, and cell signaling
- Biofilm disruption, preventing colonization and persistence of *Klebsiella* spp.

**Figure 2:**
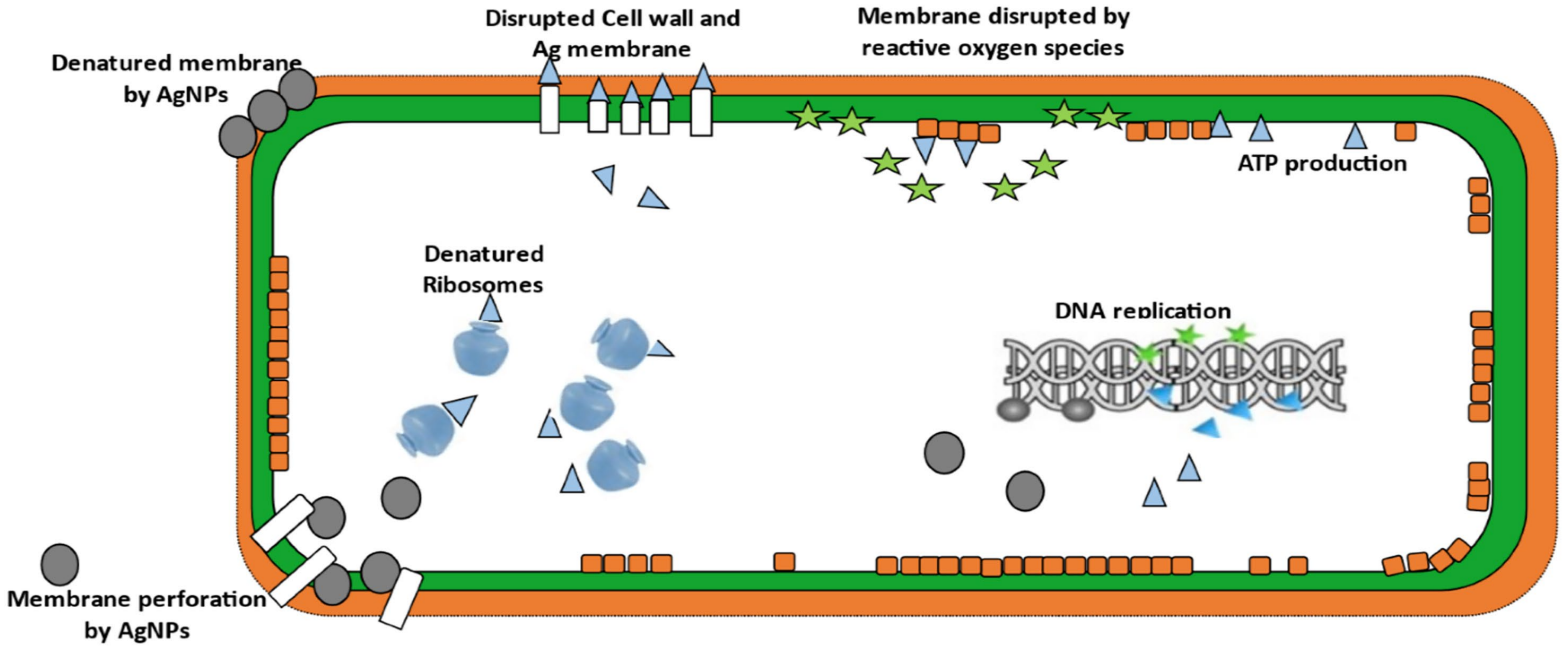
Antibacterial actions of silver nanoparticles (Ag-NPs) (Emerich and Thanos, 2024).

The small particle size (<100 nm) and high surface reactivity were consistently cited as critical factors enhancing interaction with bacterial surfaces and facilitating internalization (Negri et al., 2021; Otun et al., 2021; Mohammed et al., 2021) (Figure 2).

### Summary of findings

In summary, phytosynthesized AgNPs demonstrated consistent antibacterial activity against *Klebsiella* spp. across Africa. The strongest outcomes were linked to phenolic-rich plants, smaller nanoparticles (<50 nm), and well-characterized synthesis methods. Regional trends suggest diverse plant utilization but variable methodological rigor. Future studies should expand testing to resistant *Klebsiella* strains and explore underutilized species such as *Warburgia ugandensis*.

### Mechanistic highlights

- Allicin and ajoene from *Allium sativum* act by disrupting thiol-based enzyme systems, interfering with *Klebsiella* metabolism and reducing virulence gene expression (Ankri and Mirelman, 2020).
- Quercetin and nimbin in *Azadirachta indica* promote nanoparticle binding to bacterial walls and inhibit ATPase activity, leading to energy starvation (Singh et al., 2020)
- Sesquiterpene lactones from *V. amygdalina* (e.g., *vernodalin*) increase ROS generation, causing oxidative damage to cellular DNA and proteins (Anyanwu et al., 2021).
- Papain and flavonoids in *C. papaya* improve penetration of AgNPs into the bacterial envelope, disrupting peptidoglycan integrity (Akinmoladun et al., 2022).
- Glucosinolates from *M. oleifera* act synergistically with AgNPs by inhibiting resistance pathways such as biofilm formation and efflux pumps (Basma et al., 2020).

## Discussion

### Mechanism of action

The antimicrobial activity of silver nanoparticles (AgNPs) involves multiple mechanisms of action. AgNPs disrupt microbial cell membrane integrity, compromise membrane permeability, and cause structural damage, ultimately leading to cell death (Raza et al., 2022; Yin et al., 2021). Additionally, AgNPs generate reactive oxygen species (ROS) that induce oxidative stress, damage biomolecular structures, and contribute to microbial growth inhibition (Raza et al., 2022; Zhou et al., 2021). Furthermore, AgNPs can interrupt essential cellular processes by binding to microbial proteins and inhibiting both DNA replication and protein synthesis (Kibet et al., 2019).

**Figure 3:**
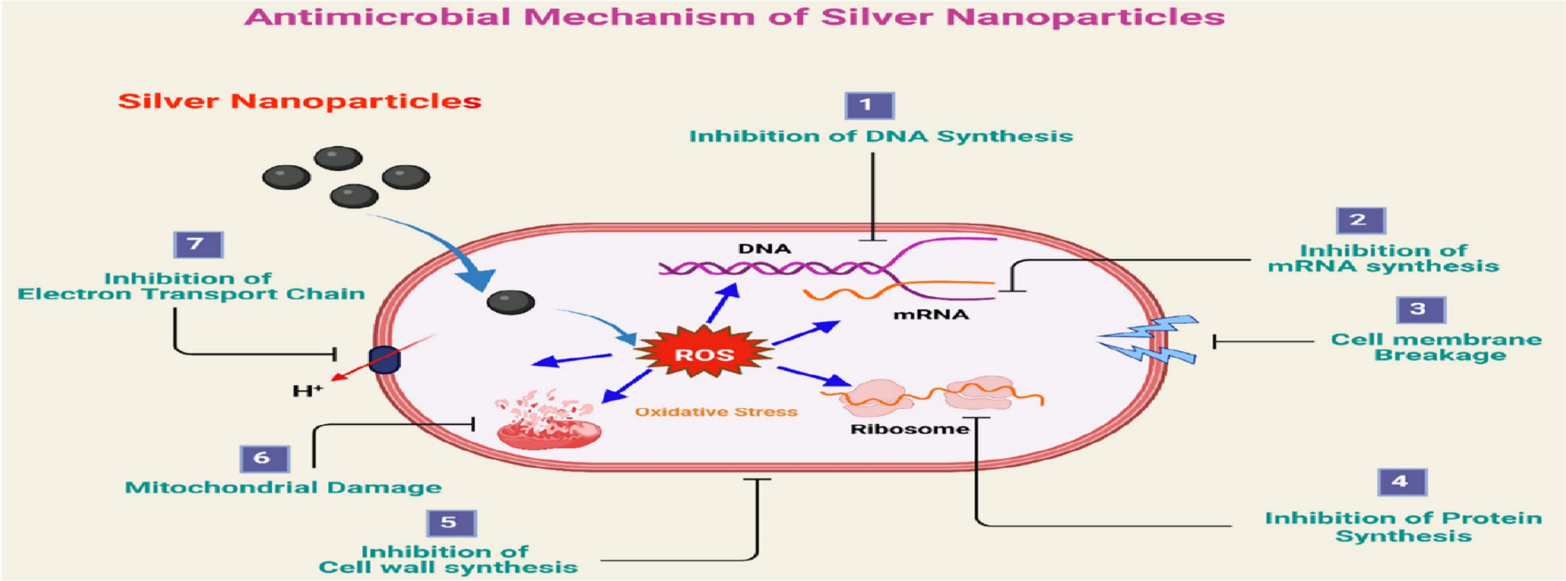
Antimicrobial mechanism of silver nanoparticles: (1) Inhibition of DNA synthesis, (2) inhibition of mRNA synthesis, (3) cell membrane destruction and the leakage of the cell constituents, (4) inhibition of protein synthesis, (5) inhibition of cell-wall synthesis, (6) mitochondrial damage, and (7) inhibition of electron transport chain (Saleh and Gaidan, 2021).

Plant phytochemicals present in the extract act as reducing and stabilizing agents during biosynthesis and may further enhance antimicrobial activity by adding synergy, increasing stability, and reducing cytotoxic effects (Oluwafemi et al., 2018) (Figure 3).

### Regional trends and strengths

The development and application of green-synthesized silver nanoparticles (AgNPs) across Africa exhibit region-specific characteristics shaped by local biodiversity, research capacity, and public health needs. These regional efforts reflect both the scientific promise and the challenges associated with phytosynthesized nanomaterials in the African context.

### North Africa

In North Africa, countries such as Egypt and Tunisia have led substantial efforts in synthesizing AgNPs using locally available medicinal plants, particularly *Carica papaya, Azadirachta indica,* and *Moringa oleifera*. Studies from this region frequently apply well-structured methodologies, including physicochemical characterization and antibacterial testing against multidrug-resistant organisms such as *Klebsiella pneumoniae* (Ahmed et al., 2020; Elsayed et al., 2023; Founou et al., 2021a,b). These nations benefit from a strong tradition of ethnobotanical research coupled with established research infrastructure, which enables the integration of traditional phytotherapy into modern nanobiotechnology.

### West Africa

In West Africa, research is flourishing in Nigeria and Ghana, where endemic species like *Vernonia amygdalina, Allium sativum,* and *Psidium guajava* are commonly utilized for nanoparticle synthesis (Adesina et al., 2021; Sanni et al., 2021; Adebayo et al., 2023). While studies from the region often demonstrate high antimicrobial efficacy, they sometimes lack standardized nanoparticle characterization protocols, which can affect reproducibility. Nonetheless, the wealth of indigenous medicinal knowledge and the abundance of bioactive plants provide a strong foundation for advancing nanomedicine in this region.

### East Africa

In East Africa, particularly Kenya and Uganda, the application of green nanotechnology is gaining momentum, supported by expanding academic networks and biotechnology programs. Plants such as *Ocimum gratissimum* and *Azadirachta indica* have been employed in the biosynthesis of AgNPs with promising results against resistant Klebsiella strains (Negri et al., 2021; Osei et al., 2021; Njoroge et al., 2024). Moreover, a regional focus on environmentally sustainable approaches and public health relevance positions East Africa as an emerging contributor to the field.

### Central Africa

While Central Africa remains underrepresented in terms of published studies, the region harbors immense unexplored phytochemical diversity. Research limitations often stem from resource constraints, limited access to advanced instrumentation, and underdeveloped nanotechnology infrastructure. Nevertheless, increasing interest in ethnopharmacology and plant-based antimicrobial discovery signals growing potential for future contributions (Ogunkunle et al., 2024).

### Southern Africa

Southern Africa, led by South Africa, stands out as one of the most advanced regions in green nanobiotechnology. The region has produced high-quality studies employing robust techniques such as UV–visible spectroscopy, X-ray diffraction, SEM, and TEM to characterize nanoparticles synthesized from *Moringa oleifera* and *Vernonia amygdalina* (Otun et al., 2021; Kamari et al., 2022). The strong institutional infrastructure, coupled with government support for nanotechnology, has enabled translation of laboratory findings into practical applications. Furthermore, emphasis on biocompatibility and cytotoxicity studies adds depth and regulatory alignment to research outputs from this region (Al-Shehri et al., 2021). Importantly, most investigations apply well-established characterization techniques such as UV–Visible spectroscopy, Transmission Electron Microscopy (TEM), and X-ray Diffraction (XRD), which are essential for confirming the physicochemical properties of synthesized nanoparticles. These methods provide critical information regarding particle size, shape, crystallinity, surface plasmon resonance, and overall stability—factors that directly influence antimicrobial performance. When combined with standardized antibacterial assays, including disk diffusion and minimum inhibitory concentration (MIC) testing, this comprehensive analytical approach enhances the methodological robustness of the studies and strengthens the reliability of the reported findings (Zhou et al., 2021; Adebayo et al., 2023). Such integrative workflows are particularly valuable for translating laboratory-scale nanoparticle research into reproducible and clinically relevant antimicrobial strategies.

### Limitations

While the data collectively highlight the potent antimicrobial activity of plant-mediated AgNPs, several limitations remain. *In vivo* validation is frequently lacking, which restricts the ability to translate these findings into clinical applications (Raza et al., 2022). Standardization of nanoparticle concentrations and methods for their preparation is another concern, adding variability across different studies (Yin et al., 2021). Furthermore, there is limited information on the cytotoxic effects and biocompatibility of these nanoparticles, which must be thoroughly investigated before proceeding with human and veterinary applications (Al-Shehri et al., 2021).

### Future directions

Despite the increasing interest in green synthesis of silver nanoparticles using African medicinal plants, no study to date has reported the use of *Warburgia ugandensis*, a plant known for its potent antimicrobial compounds, for synthesizing AgNPs against *Klebsiella* spp. This represents a significant knowledge gap, especially given the urgent need to discover novel, plant-based antimicrobial agents to combat multidrug-resistant pathogens. Future research should focus on exploring *W. ugandensis* as a potential phytochemical source for nanoparticle synthesis and testing its efficacy against resistant *Klebsiella* strains.

Establishing standardized protocols for nanoparticle synthesis, characterization, and antimicrobial testing will be critical to ensure consistency, comparability, and reproducibility across studies. Additionally, comprehensive *in vivo* studies and clinical evaluations are essential to determine the safety, pharmacokinetics, and therapeutic potential of these nanoparticles in real-world settings. Long-term assessments of their environmental impact and biocompatibility will also be necessary to inform regulatory frameworks and promote responsible integration into healthcare and biotechnology sectors.

### Potential applications of green-synthesized silver nanoparticles

The green synthesis of silver nanoparticles presents a promising avenue for addressing the escalating global burden of antimicrobial resistance. Owing to their potent antibacterial properties, plant-mediated silver nanoparticles are strong candidates for incorporation into a variety of biomedical applications. These include antimicrobial wound dressings, dental materials, and surface coatings for catheters, implants, and other medical devices prone to microbial colonization.

Beyond clinical settings, their utility extends to industrial and environmental domains. These nanoparticles may be employed in food packaging to limit microbial contamination and extend shelf life, as well as in water purification systems to control pathogenic microorganisms and promote public health. Their inherent advantages—biocompatibility, low production cost, and eco-friendly synthesis—support scalable and sustainable deployment.

To fully harness these applications, further research is needed to address critical gaps in toxicological assessment, *in vivo* efficacy, and production standardization. Ensuring the reproducibility and safety of these nanomaterials will be vital for their successful transition into regulated healthcare and industrial markets.

## Conclusion

Plant extract–mediated silver nanoparticles exhibit consistent and broad-spectrum antibacterial activity against *Klebsiella* spp., as demonstrated by numerous *in vitro* studies conducted across Africa. This biogenic approach offers a sustainable and cost-effective alternative to traditional antimicrobial agents, leveraging the reducing and stabilizing capabilities of plant-derived phytochemicals to produce nanoparticles with optimal physicochemical and functional properties.

These green-synthesized nanoparticles hold considerable promise as stand-alone or adjunct therapeutic options in the management of multidrug-resistant bacterial infections. Their potential extends beyond laboratory settings, indicating applicability in diverse biomedical and environmental contexts. However, despite these encouraging findings, several critical challenges must be addressed to support clinical translation. These include rigorous toxicological evaluations, in vivo validation, and the establishment of standardized synthesis and testing protocols.

Advancing this field will require coordinated multidisciplinary efforts that integrate nanotechnology, microbiology, pharmacology, and public health. By ensuring both safety and efficacy, plant-mediated silver nanoparticles could become a pivotal tool in the next generation of antimicrobial strategies, particularly in resource-limited settings where conventional treatments are often inaccessible or ineffective.

## Data Availability

The original contributions presented in the study are included in the article/Supplementary material, further inquiries can be directed to the corresponding author/s.

## References

[ref2] Adebayo S. O. Adeoye A. O. Lawal A. O. . (2023). Silver nanoparticles synthesized from *Psidium guajava* extract exhibit strong antimicrobial activity against ESBL-producing *Klebsiella pneumoniae*. Niger. J. Basic Appl. Sci. 31, 52–60.

[ref3] Adesina O. A. Akinyemi O. O. Oladipo I. A. (2021). Biosynthesis of silver nanoparticles using *Allium sativum* and evaluation of their antibacterial potential against *Klebsiella* species. Afr. J. Biotechnol. 20, 250–258. doi: 10.5897/AJB2021.17087

[ref4] Adongo J. O. Muturi M. W. Njogu P. M. (2023). Phytochemical and pharmacological significance of *Warburgia ugandensis*: a review. J. Ethnopharmacol. 308:116334.

[ref5] Ahmed R. A. El-Banna H. A. Elnaggar M. E. (2020). Eco-friendly synthesis of silver nanoparticles using *Carica papaya* leaf extract and their antibacterial activity. Environ. Nanotechnol. Monit. Manag. 14:100353. doi: 10.1016/j.enmm.2020.100353

[ref6] Akinmoladun F. O. Oloyede S. O. Ogundele O. M. . (2022). Characterization and antibacterial analysis of *Carica papaya*-based AgNPs. Sci. Afr. 15:e01122.

[ref7] Al-Shehri S. Ali A. Moin A. . (2021). Green synthesis of metal nanoparticles using plant extracts: applications in antimicrobial and biomedical fields. Antioxidants (Basel) 10:1885.34942988

[ref8] Ankri S. Mirelman D. (2020). Antimicrobial properties of allicin. Microbes Infect. 22, 233–240. 10.1016/s1286-4579(99)80003-3 10594976

[ref9] Anyanwu M. U. Onuigbo H. C. Anosike I. J. . (2021). Pharmacological actions of *Vernonia amygdalina* in MDR infections. BMC Complement. Med. Ther. 21:220.34479568

[ref10] Basma A. A. Khalil H. Mustafa M. . (2020). Phytochemical and biological activities of *Moringa oleifera* extracts. Plants. 9:1169.32916928

[ref11] Durán N. Seabra A. B. Fávaro W. J. Nakazato G. (2016). Mechanistic aspects in the biogenic synthesis of extracellular metal nanoparticles by peptides, bacteria, fungi, and plants. Appl. Microbiol. Biotechnol. 100, 6555–6570. 21484205 10.1007/s00253-011-3249-8

[ref12] Elsayed A. M. Khalil M. A. Hassan H. M. (2023). Antibacterial activity of silver nanoparticles synthesized from *Moringa oleifera* leaf extract against clinically isolated *Klebsiella* spp. Microb. Pathog. 168:105620. doi: 10.1016/j.micpath.2023.105620

[ref9001] Emerich D. F. Thanos C. G. (2024). Nanotechnology and medicine. Expert Opinion on Biological Therapy, 3, 655–663. doi: 10.1517/14712598.3.4.655 12831370

[ref13] Founou L. L. Founou R. C. Essack S. Y. (2021a). Antibiotic resistance in food production and the environment: a systematic review of the literature from Africa. Antibiotics (Basel) 10:304.33809460

[ref14] Founou L. L. Founou R. C. Essack S. Y. (2021b). Antibacterial potential of green synthesized silver nanoparticles from *Allium sativum* against multidrug-resistant *Klebsiella* spp. Front. Microbiol. 12:695643. doi: 10.3389/fmicb.2021.695643

[ref15] Franci G. Falanga A. Galdiero S. Palomba L. Rai M. Morelli G. . (2015). Silver nanoparticles as potential antibacterial agents. Molecules 20, 8856–8874. doi: 10.3390/molecules20058856, 25993417 PMC6272636

[ref17] Kamari A. Mokone M. E. Dlamini P. (2022). Synthesis and characterization of silver nanoparticles using *Moringa oleifera* and their antimicrobial activities against gram-negative bacteria. S. Afr. J. Sci. 118, 1–8. doi: 10.17159/sajs.2022/10168

[ref18] Kibet J. Ochieng R. Otieno F. . (2019). Antibacterial activity of silver nanoparticles synthesized using *Ocimum gratissimum* extract. East Afr. Med. J. 96, 443–450.

[ref19] Marambio-Jones C. Hoek E. M. V. (2010). A review of the antibacterial effects of silver nanomaterials and potential implications for human health and the environment. J. Nanopart. Res. 12, 1531–1551. doi: 10.1007/s11051-010-9900-y

[ref9002] Mcarthur A. Stephenson M. Aromataris E. (2020). Introduction to the JBI critical appraisal tool. 2127–2133. doi: 10.11124/JBISRIR-D-19-00099 33038125

[ref20] Mohammed A. Oluwafemi S. Bamidele T. . (2021). Biosynthesis and characterization of silver nanoparticles using *Euphorbia hirta* and their antibacterial effect against *Klebsiella pneumoniae*. Int. J. Nanomedicine 16, 3255–3266.34012260

[ref9003] Morante N. Folliero V. Annunziata F. D. Capuano N. Mancuso A. Monzillo K. . (2024). Characterization and Photocatalytic and Antibacterial Properties of Ag- and TiO. 10.3390/ma17102178 PMC11122886 38793245

[ref21] Musyoka T. M. Odhiambo J. A. Omwenga E. O. (2020). Antimicrobial and cytotoxic activities of *Warburgia ugandensis* extracts. BMC Complement. Med. Ther. 20:155.32448223

[ref9004] Navon A. Machlev R. Carmon D. Onile A. E. Belikov J. Levron Y. (2021). Effects of the COVID-19 Pandemic on Energy Systems and Electric Power Grids — A Review of the Challenges Ahead. 1–14.

[ref22] Negri M. Mwangi J. N. Njoroge J. M. (2021). Green synthesis of silver nanoparticles using *Azadirachta indica* and their antibacterial efficacy against *Klebsiella pneumoniae*. Nano 11:2345. doi: 10.3390/nano11092345

[ref23] Njogu P. M. Waweru M. M. Muturi M. W. (2022). Phytochemistry, pharmacology, and conservation of *Warburgia ugandensis*: a mini-review. S. Afr. J. Bot. 148, 298–306.

[ref24] Njoroge P. K. Otieno D. O. Mwangi P. W. (2024). Antibacterial potential of silver nanoparticles synthesized with *Vernonia amygdalina* extract against clinical isolates of *Klebsiella* spp. J. Nanomed. 15, 101–110. doi: 10.2147/JNAN.S350987

[ref25] Ogunkunle C. Adebayo S. O. Fadeyi O. O. . (2024). Green synthesis of silver nanoparticles using plant extract: a systematic review of antimicrobial mechanisms. Antimicrob. Resist. Infect. Control 13:42.38616284

[ref26] Okello S. V. Omondi A. Onyango P. (2020). Traditional and contemporary uses of *Warburgia ugandensis* in East Africa. J. Med. Plants Res. 14, 221–230.

[ref27] Oluwafemi O. S. Mbatha J. Khanyile M. . (2018). Green synthesis of silver nanoparticles using extracts of plants from the eastern cape of South Africa. Mater. Lett. 187, 111–114.

[ref28] Omwenga E. O. Muturi M. W. Musyoka T. M. (2021). Antifungal and antioxidant properties of drimane sesquiterpenes from *Warburgia ugandensis*. Front. Pharmacol. 12:667892.

[ref29] Osei P. Boateng B. Oduro-Yeboah D. . (2021). Biosynthesized silver nanoparticles from *Azadirachta indica* against multidrug-resistant *Klebsiella pneumoniae*. Ghana J. Sci. 61, 92–100.

[ref30] Otun S. O. Mbatha J. Nkosi T. (2021). Characterization and antimicrobial activity of silver nanoparticles synthesized from *Vernonia amygdalina* leaf extract. J. Appl. Microbiol. 130, 1135–1143. doi: 10.1111/jam.14817

[ref200] Page M. J. Mckenzie J. E. Bossuyt P. M. Boutron I. Hoffmann C. Mulrow C. D. . (2021). The PRISMA 2020 statement: an updated guideline for reporting systematic reviews Systematic reviews and Meta-Analyses. doi: 10.1136/bmj.n71 PMC8008539 33781348

[ref9005] Rahman A. Ahmed M. Khan H. Kamel N. A. (2024). Journal of King Saud University - Science Beta maritima mediated silver Nanoparticles: Characterization and evaluation of Antibacterial, Antifungal, and antioxidant activities. Journal of King Saud University - Science. 36:103219. doi: 10.1016/j.jksus.2024.103219

[ref33] Raza K. Ansari M. Bhattacharya S. . (2022). Antimicrobial mechanisms of metal nanoparticles and their plant-mediated biosynthesis. Front. Microbiol. 13:943967.

[ref9006] Saleh R. F. Gaidan A. M. (2021). Biosynthesis and characterization of silver nanoparticles using Cinnamomum zeylanicum extract and a study of antibacterial effect against multi-drug resistance Gram-negative bacteria. Biomedicine (India), 41, 249–255. doi: 10.51248/.v41i2.791

[ref35] Sanni O. A. Oladejo S. O. Adesanya O. A. (2021). Green synthesis of silver nanoparticles from *Carica papaya* and their antibacterial activity against *Klebsiella* species. Heliyon 7:e06715. doi: 10.1016/j.heliyon.2021.e06715 33898834 PMC8056424

[ref36] Singh R. Singh S. Dwiwedi S. . (2020). *Azadirachta indica* phytochemicals in antibacterial and nanotherapeutic applications. J. Ethnopharmacol. 263:113256.

[ref39] World Health Organization (WHO). (2017). Global priority list of antibiotic-resistant bacteria to guide research, discovery and development of new antibiotics. Available online at: https://www.who.int/publications/i/item/global-priority-list-of-antibiotic-resistant-bacteria

[ref40] Yin L. Liu Y. He X. . (2021). Biosynthesis of silver nanoparticles by plant extract and their antimicrobial mechanisms against drug-resistant pathogens. Antimicrob. Agents Chemother. 65, e01904–e01920.

[ref41] Zhou L. Wang Y. Liu J. . (2021). Antibacterial mechanisms of silver nanoparticles against gram-negative pathogens. Front. Chem. 9:813967.

[ref9007] Zhou K. Xue C. Xu T. Shen P. Wei S. Wyres K. L. . (2023). A point mutation in recC associated with subclonal replacement of carbapenem- resistant Klebsiella pneumoniae ST11 in China. 1–14. doi: 10.1038/s41467-023-38061-z PMC10147710 37117217

