# Supplementary material for: Antibacterial activity of plant-extract mediated silver nanoparticles against Klebsiella spp. in Africa: a systematic review
Source: Front Microbiol. 2026 Jan 12;16:1673235. doi: 10.3389/fmicb.2025.1673235 (PMC12833210; doi:10.3389/fmicb.2025.1673235)
Supplement: Supplementary file 1 [file Data_Sheet_1.pdf]

Supplemental Table 1. Detailed Characteristics and Antibacterial Outcomes of Included Studies (2015–2025)

Author (Year)	Country	Plant species	NP size (nm)	Assay(s)	Zone of inhibition (mm)	MIC (µg/mL)	MBC (µg/mL)	Target strain
Ahmed et al., 2020	Egypt	*Albizia coriaria*	NR	Disk, MIC	20	25	NR	*K. pneumoniae*
Otun et al., 2021	South Africa	*Vernonia amygdalina*	30–60	Disk, MIC	18	12.5	NR	*K. pneumoniae*
Adebayo et al., 2023	Nigeria	*Aspilia africana*	NR	Disk, MIC	22	10	20	*K. pneumoniae*
Mohammed et al., 2021	Nigeria	*Euphorbia hirta*	30–60	Disk, MIC	16	12.5	NR	*K. pneumoniae*
Kinyua et al., 2020	Kenya	*Senna alata*	25–55	MIC, MBC	19	6.25	12.5	*Klebsiella spp.*
Akinlabi et al., 2023	Ghana	*Ocimum gratissimum*	30–50	Disk, MIC	22	18.75	NR	*K. pneumoniae*
Adesina et al., 2021	Nigeria	*Annona senegalensis*	NR	Disk, MIC	17	20	40	*Klebsiella spp.*
Kamari et al., 2022	South Africa	*Moringa oleifera*	NR	Disk, MIC	23	15.5	NR	*K. pneumoniae*
Elsayed et al., 2023	Egypt	*Securidaca longepedunculata*	NR	Disk, MIC	20	25	NR	*K. pneumoniae*
Njoroge et al., 2024	Kenya	*Mondia whitei*	NR	Disk, MIC	18	10	NR	*K. pneumoniae*
Ogunkunle et al., 2024	Nigeria	*Tithonia diversifolia*	NR	Disk, MIC	15	25	50	*K. pneumoniae*
Negri et al., 2021	Uganda	*Aloe vera*	NR	Disk, MIC	21	9.5	NR	*Klebsiella spp.*
Founou et al., 2021	Tunisia	*Allium sativum*	NR	Disk, MIC	19	8	16	*Klebsiella spp.*

Author (Year)	Country	Plant species	NP size (nm)	Assay(s)	Zone of inhibition (mm)	MIC (µg/mL)	MBC (µg/mL)	Target strain
Sanni et al., 2021	Nigeria	*Harpagophytum procumbens*	NR	Disk, MIC	14	22	NR	*K. pneumoniae*

Abbreviations: NP – nanoparticle; MIC – Minimum Inhibitory Concentration; MBC – Minimum Bactericidal Concentration; NR – Not reported.

SUPPLEMENTAL TABLE 2: JBI Risk of Bias Assessment Summary

No.	Study (Author, Year)	Objective Clarity	Design Fit	Material Detail	NP Characterization	Control Use	Standardized Assay	Result Clarity	Replicability	Risk Level
1	Ahmed et al., 2020	Yes	Yes	Yes	Yes	Yes	Yes	Yes	Yes	Low
2	Otun et al., 2021	Yes	Yes	Yes	Yes	Yes	Yes	Yes	Yes	Low
3	Adebayo et al., 2023	Yes	Yes	Yes	Yes	Yes	Yes	Yes	Yes	Low
4	Mohammed et al., 2021*	Yes	Yes	Yes	Partial	Yes	Yes	Yes	Moderate	Moderate
5	Kinyua et al., 2020*	Yes	Yes	Yes	Yes	Partial	Yes	Yes	Partial	Moderate
6	Akinlabi et al., 2023*	Yes	Yes	Yes	Yes	Yes	Yes	Partial	Partial	Moderate
7	Adesina et al., 2021	Yes	Yes	Yes	Partial	Yes	Yes	Yes	Partial	Moderate
8	Kamari et al., 2022	Yes	Yes	Yes	Yes	Yes	Yes	Yes	Yes	Low
9	Elsayed et al., 2023	Yes	Yes	Yes	Yes	Yes	Yes	Yes	Yes	Low

No.	Study (Author, Year)	Objective Clarity	Design Fit	Material Detail	NP Characterization	Control Use	Standardized Assay	Result Clarity	Replicability	Risk Level
10	Njoroge et al., 2024	Yes	Yes	Yes	Yes	Partial	Yes	Yes	Yes	Moderate
11	Ogunkunle et al., 2024	Yes	Yes	Yes	Partial	Yes	Yes	Yes	Yes	Moderate
12	Negri et al., 2021	Yes	Yes	Yes	Yes	Yes	Yes	Yes	Yes	Low
13	Founou et al., 2021	Yes	Yes	Yes	Yes	Yes	Yes	Yes	Yes	Low
14	Sanni et al., 2021	Yes	Yes	Yes	Partial	Yes	Yes	Yes	Partial	Moderate
